# Facile strategy toward the development of novel binder and thickening agent from apple rock bael for textile printing

**DOI:** 10.1038/s41598-025-11544-3

**Published:** 2025-07-28

**Authors:** N. S. Elshemy, S. H. Nassar, Nancy S. Elhawary, Mona M. Ali

**Affiliations:** 1 Department of Dyeing and Textile Printing, Textile Institute, National Research Centre, Giza, Egypt; 2 Faculty of Women for Arts, Science and Education, Ain Shams University, Cairo, Egypt; 3 Department of Dyeing, Printing, and Textile Auxiliaries, Textile Research and Technology Institute, National Research Centre, 33 El-Buhouth Street, Dokki, P.O. Box 12622, Cairo, Egypt

**Keywords:** Microwave irradiation, Textile printing, Binder, Thickening agent, Biotechnology, Environmental sciences, Environmental social sciences

## Abstract

This study focuses on isolating a natural binder and thickening agent derived from the Aegle marmelous fruit. The isolated natural gum can be effectively employed as a binder and thickening agent in fabric printing applications. The findings indicate that this natural gum has remarkable rheological characteristics, essential for achieving optimal printing results. Microwave irradiation techniques and thermal bonding, varying in duration, power, and temperature, were employed to fix the printed samples that utilized the isolated natural gum. The results demonstrated that printed textiles exhibited excellent color fastness, with samples treated via microwave fixation showing enhanced color saturation, as evidenced by higher K/S values. A thorough evaluation of the physical and mechanical properties was conducted, including assessments of color yield, uniformity, absorption, and fixing efficiency. The results indicate that both weight loss and water absorption tend to increase over time. The natural gum isolated from Aegle marmelous shows minimal loss and absorption, in contrast to commercial gum (Sodium alginate), which exhibits significantly higher levels. Scanning electron microscopy (SEM) highlights distinct differences in particle morphology between the two types of gum; the commercial variety presents spherical aggregates, while the isolated natural gum features elongated thread-like particles. Extended microwave exposure leads to enhanced color intensity, which is influenced by the fabric structure and type of gum used. The K/S value peaks at 70 watts and subsequently decreases at 90 watts for printed cotton and cotton/polyester blends, while printed wool achieves the best results at 50 watts for 60 s. Closed samples consistently show enhanced K/S values, irrespective of the microwave settings. Regarding thermo fixation, as fixation temperatures and duration increase, K/S values typically rise, except for printed cotton. The K/S values reached their maximum at 160 °C for 6 min for wool and polyester/cotton blends, whereas cotton peaked at 140 °C under the same conditions. The observed variations in color yield, penetration, and fixation percentages among the different fabrics are attributed to their unique chemical compositions and characteristics, as well as the effects of microwave irradiation. Furthermore, employing pulsed microwave irradiation helps regulate temperature and mitigate exothermic reactions, resulting in improved dye-fabric interactions and overall stability of the dyeing process. This thorough analysis highlights the potential of utilizing natural agents derived from Aegle marmelous in contemporary textile printing, supporting sustainable practices while upholding performance standards.

## Introduction

The rise in demand for environmentally sustainable binders and thickening agents within the textile printing sector can be attributed to advancements in technology. Traditionally, synthetic chemical thickening has been employed in textile processing, which unfortunately can lead to the generation of hazardous byproducts such as nitrosamines, azo dyes, and various acidic compounds, posing threats to both ecological balance and human health^1–5^. By integrating eco-friendly, non-toxic binders and thickening agents into textiles, the formation of these harmful substances can be mitigated, thereby fostering a shift towards materials derived from less harmful sources, including those of plant and animal origins. Moreover, the incorporation of cellulose into textiles has been recognized as a significant enhancer of the optical properties of fabrics, leveraging its capacity to improve overlap characteristics^6–9^. Recent studies have explored the potential of natural materials as binders and thickening agents within the textile domain. Despite these advancements, there remains a gap in extraction methodologies designed to effectively evaluate the qualitative potential of valuable cellulose content in these materials. Notably, in various regions, the fruits of Aegle marmelous have gained attention as a viable natural resource, containing substantial carbohydrate quantities^10–14^.

In recent years, manufacturers in least developed and emerging nations have prioritized producing low-cost, value-added products while complying with sustainability standards. The textile and garment industries face social, economic, and environmental challenges in adopting environmentally friendly practices, leading to a loss of cost advantages. The garment industry significantly harms the environment due to high energy and water use and the employment of harmful dyes and chemicals. Additionally, business operations often generate excessive pollution and waste. The fast fashion trend, seen in retailers like Walmart and GAP, contributes to increased consumer waste. Despite these challenges, there is a growing global emphasis on social, economic, and environmental responsibility among consumers. Clothing produced under slow fashion trends uses recyclable, eco-friendly materials aimed at sustainable production, though meeting these standards can be difficult. The ethical beliefs of producers and consumers are crucial in this transition. Addressing the ecological and financial damage from the garment sector demands substantial time and resources^15–21^.

This study focused on the potential benefits and properties associated with utilizing Aegle marmelous fruits, alongside their innovative hydro-metallurgical extraction process, which presents a promising alternative to conventional chemical approaches in textile printing. It is necessary to produce textiles with less harmful outputs to defend the global environment. Environmentally friendly materials are replacing those with highly toxic substances^22–26^. However, we still need more information to establish the most comprehensive classification of natural materials for printing processes. Furthermore, using a combination of binders and thickening agents from the same source represents a significant innovation in studying commercially interesting fields such as technology, science, and economics.

Textile industries can utilize various benefits of materials to create more unique and attractive fabrics, and the use of large-scale thickening directly leads to cheaper textiles. Filling the gap with currently missing research objectives would contribute to new environmental directions with valuable natural resources^27–31^. Furthermore, providing information on the historical use of medicinal plants can enhance the overall utility of thickening and add unique value to the assisted textiles. Thickening happens when the substrate area extends. It resists three types of cotton fabric abrasion, tenacity, and elongation testing. It may be convenient to transfer^32–36^. A binder, containing hydroxyethyl cellulose, sodium alginate, and gum, can be used within a suitable printing machine, providing antimicrobial benefits and incorporating thickening from natural resources into textiles. Cotton fabric handles also assess their wetness, stiffness, hardness, smoothness, thickness, softness, and flexibility.

All previous studies were conducted to study the use of extracted materials as a thickener only, but their use as an alternative to thickeners and binders was not studied. So, this research aims to extract the resinous substance found in the Aegle marmelous and the possibility of using it as an alternative to thickener and or a binder in textile printing.

## Methods

### Fabrics

Miser Co. supplied mill scoured, and mercerized plain-weave cotton fabric and 60:40 polyester/cotton blend fabric (spinning and weaving in El Mahalla El-Kobra, Egypt).

Miser Co. provided 100% wool fabrics, which had undergone mill-scouring to spin and weave.

To achieve the removal of contaminants, a methodical washing process was implemented as follows:

$$\:\text{C}\text{o}\text{t}\text{t}\text{o}\text{n}\:\text{f}\text{a}\text{b}\text{r}\text{i}\text{c}\text{s}+\:3\text{g}/\text{l}\text{n}\text{o}\text{n}\:\text{i}\text{o}\text{n}\text{i}\text{c}\:\text{d}\text{e}\text{t}\text{e}\text{r}\text{g}\text{e}\text{n}\text{t}\:\left(\text{H}\text{o}\text{s}\text{t}\text{a}\text{p}\text{a}\text{l}\:\text{C}\text{V}\right),+\:3\text{g}/\text{l}{\text{N}\text{a}}_{2}\:\text{C}{\text{O}}_{3}\xrightarrow{{90}^{^\circ\:\:\:}\text{C},\:60\text{m}\text{i}\text{n}.}\:\text{C}\text{o}\text{l}\text{d}\:{\text{H}}_{2}\text{O}\:\xrightarrow{\text{d}\text{r}\text{i}\text{e}\text{d}.\:}\text{a}\text{t}\:\text{r}\text{o}\text{o}\text{m}\:temp$$

$$\:Polyester/Cotton+5g/l\:{Na}_{2}C{O}_{3\:}\left(Sodium\:cabonate\right)\xrightarrow{{50}^{^\circ\:\:\:}C,\:\:\:30\:min}\:Cold\:{H}_{2}O\xrightarrow{dried}\:at\:room\:temp$$

Aegle marmelouse were purchased from the commercial market, Cairo, Egypt. The botanical specimen Aegle marmelos, referred to as bael, was first systematically categorized and documented by botanist Lorenzo Benoît Correa. He designated the species as Aegle marmelos (L.) Corrêa, where the ‘L.’ signifies the prior classification established by Linnaeus. Correa’s findings were disseminated in the early 19th century. This plant is available in herbal shops in our country and can be obtained ripe in July and June (37).

### Chemicals

Nonionic detergent (Hostapal CV), characterized as an anionic textile auxiliary derived from alkyl aryl-polyglycol ether.

Sodium alginate used as a commercial thickener derived from Clarient.

Other chemicals (sodium bicarbonate, acetic acid, and sodium dihydrogen phosphate are classified as laboratory-grade chemicals.

### Gum extraction from Aegle marmelous fruits

Analyzing the process of gum isolated from the fruits of Aegle marmelous reveals a significant interplay between the biochemical constituents of the fruit and the methodologies employed in the extraction process. The Aegle marmelous, commonly known as bael fruit, contains various polysaccharides and other compounds that facilitate the production of gum. The extraction typically involves methods such as solvent extraction or mechanical means to isolate the gum from the fruit’s pulp and other materials. This process necessitates a careful consideration of factors such as temperature, solvent type, and extraction time, all of which can influence the yield and quality of the gum obtained. Understanding these aspects is crucial for optimizing extraction techniques and ensuring the purity of the final product for potential applications in the food, pharmaceutical, and industrial sectors^38–43^.

**Fig. 1:**
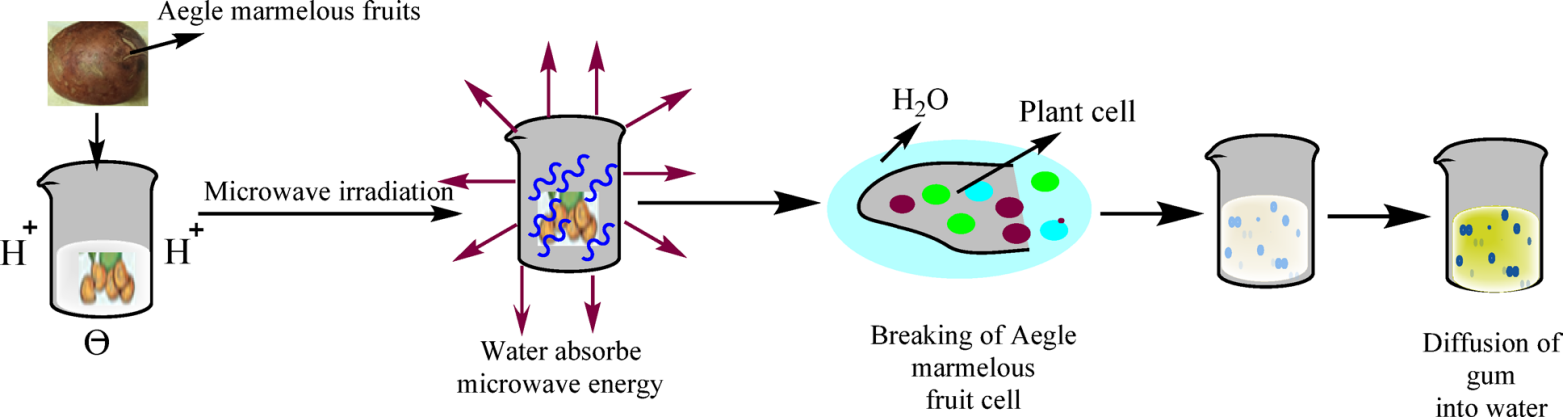
Microwave-assisted gum extraction from Aegle marmelous fruit.

Collected bael fruits, were partially ripe and subsequently bisected after the hard pericarp was broken. The amber-colored, dense, and sticky translucent gummy material, along with seeds and pulp, was mashed in a 2% v/v glacial acetic acid solution (as a catalyst) to create a slurry. This mixture was then boiled in a water bath for 10 min and left to rest overnight. The slurry was filtered using muslin cloth to eliminate debris. Excess acetone (as a precipitating agent) was utilized to precipitate the gum. The residual solvent removed By evaporation and washing. The gum was dried in a vacuum oven at 50 °C and ground to yield a light brown powder (Fig. 1)^44^.

### Coloring

**Fig. 2:**
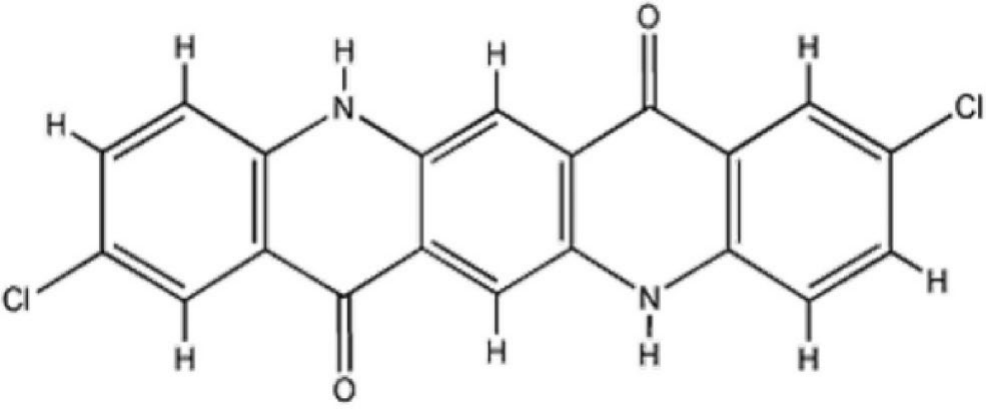
Chemical configuration of Coloring Red 210. (Source: www.dyestuffintermediates.com)

Pigment Red 210 (Fig. 2).

### Preparation of the printing paste

The printing paste was crafted independently using the extracted gum functioning as both a binder and thickening agent, thereby eliminating the necessity for additional stiffening or thickening components. The formulation details used to produce the printing paste are documented in Table 1^45,46^.

**Table 1:** Formulation of the printing paste.

Components	Weight/gm
pigment	20
Thickener	80
Urea	10
Water	X
Sodium dihydrogen phosphate	10
Total	1,000 g

### Printing technique

Using a manual flat silk screen printing technique, all the samples were meticulously washed with cold water after the fixation process and dried at room temperature.

### Fixation methods

Under the effect of microwave irradiation at different Watts and time/sec (cover or leave it off).

Thermo fixation at different temperature/°C and different times/minutes.

All the samples subjected to fixation undergo a washing process (2 g/l, L.R 1:50, at 60 °C for 10 min).

### Analysis and measurements

#### Rheological measurement

Rheological and apparent viscosity were conducted utilizing a 9Brookfield DV-111 Programmable Rheometer (USA) across a range of shear rates from 3.4 to 68 s⁻¹) at 25 °C (Eq. 1)^47,48^.

1 $$\eta\:\left(apparent\:viscosity\:in\:poise\right)=\frac{t\:\left(sharing\:stress\right(dyne/{cm}^{2})}{D\:\left(rate\:of\:share\:\right({S}^{-1})}$$

#### The water absorbency of extracted natural gum

1. Generated extracted gum films by placing the thickening material in Petri dishes,
2. Dry in the air.
3. Cured (for 4 min) at (160 °C), then cooled overnight at room temperature.
4. Weight of each thickening film.
5. Each films were immersed in distilled water for (6, 12, 24, and 48 h) at room temperature.
6. Removed excess surface water using filter paper, and reweighed the swollen films.
7. Calculated the water absorbency (Eq. 2)^49^.

2 $$\:Water\:absorbence\:\left(\%\right)=\:\frac{{W}_{1}\left(weight\:of\:the\:swollen\:sample\right)}{W\:\left(weight\:of\:the\:original\:sample\right)}\times\:100$$

#### Weight loss of extracted natural gum

The natural gum films were generated utilizing the method outlined above. These films underwent a curing process lasting 4 min at a temperature of 160 °C, after which they were allowed to cool at room temperature for 24 h. The weight of the films was subsequently recorded. Following this, the films were subjected to treatment with distilled water for varying time intervals of 24 and 48 h. After the treatment, a drying phase at room temperature was implemented for an additional 24 h, and the films were reweighed. The resulting weight loss was determined using Eqs. 3^50,51^.

3 $$\:Weight\:loss\:\left(\%\right)=\:\frac{{W}_{2}\left(dry\:weight\:of\:the\:film\:after\:treatment\right)}{W\left(dry\:weight\:of\:the\:film\:before\:treatment\right)}\times\:100$$

#### Color measurements

Color strength (K/S) of all printed and cured was conducted at a specific wavelength (525 nm). By employing reflectance measurements with the Perkin–Elmer (Lambda 3 B) spectrophotometer equipped with pulsed xenon lamps as a light source (Ultra Scan Pro, Hunter Lab, USA), Eq. (4)^48–52^.

4 $$\:\raisebox{1ex}{$K$}\!\left/\:\!\raisebox{-1ex}{$S$}\right.=\frac{{\left(1-R\right)}^{2}}{2R}-\frac{{\left(1-{R}_{0}\right)}^{2}}{2{R}_{0}}$$

where K is the absorption coefficient; S is the dispersion coefficient; and R is the reflectance of the cloth at its maximum wavelength^49^.

#### Color yield

The K/S values from each printed sample were assessed at three separate points, ensuring the absence of folds, utilizing a Perkin-Elmer (Lambda 3 B) visible spectrophotometer. The samples underwent analysis from the top and bottom across both surfaces (Eq. 5)^51,52^

5 $$\:Penetration\:\left(\%\right)=\:\frac{{\left(K/S\right)}_{1}\left(color\:strength\:of\:the\:face\:side\right)}{\frac{\left[\left({K/S}_{1}\right)+\:{\left(K/S\right)}_{2}\left(color\:strength\:of\:the\:bottom\:side\right)\right]}{2}}\times\:100$$

#### Colorfastness

The assessment of colorfastness was performed under standardized methods (AATCC 08-2007) and AATCC 61-2009^50–54^.

#### Bio-degradation study

The biodegradability of isolated gum samples was examined using the soil/compost burial method, which involved a 50:50 weight ratio. Soil sourced from Cairo, Egypt, was carefully placed into pots. The isolated gums are drying at 40 °C for 12 h. Subsequently, 20 individual isolated gum samples (0.50 g) were buried in separate pots at a depth of 3 centimeters. The pots were kept at ambient temperature and were irrigated every three days with 50 milliliters of water. The weight of each isolated gum sample was measured every seven days throughout 7 weeks. After each interval, the samples were washed with water and dried at 40 °C for an additional 12 h. The weight, represented as a percentage of weight loss, was recorded over time and used to calculate the percentage of biodegradability using specific Eq. 6.

6 $$\:Biodegradability\%=\frac{Initial\:weight\:of\:sample\:-\:Final\:weight\:of\:sample}{Final\:weight\:of\:sample}\times\:100$$

### Statistical analysis

All experiments and analyses were conducted a minimum of three times each. For the statistical analysis, various methods were employed, including analysis of variance (ANOVA) and Duncan’s test for mean significance. These analyses were performed at a significance level of *p* < 0.05 using SAS software version 9.

## Results

### Rheological characteristics of extracted natural gum as a viscosity enhancer (Thickening Agent)

Making printed fabric starts with keeping a close eye on the amount of gum in the printing paste. How this paste behaves, like its makeup and thickness, really affects how it flows, which is super important for printing. When we print, applying pressure makes the polymer chains in the paste shift around, which not only makes it thinner but also helps it flow better. Plus, the binder we pick matters a lot; it plays a key role in setting the paste’s thickness and how well it mixes with everything else. All these different factors come together to decide how good the final printed samples turn out. To see how well gum-based printing pastes work, we took a look at their thickness levels^1,55^.

**Fig. 3:**
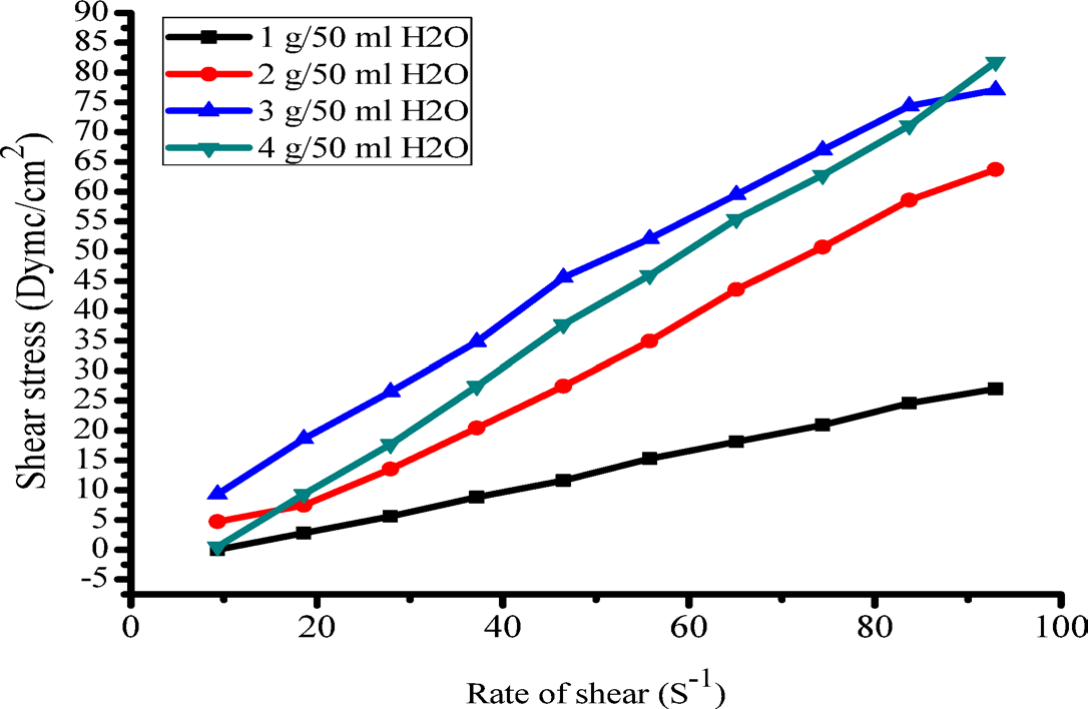
Shows how different amounts of extracted gum affect the flow and texture of printing paste.

The viscosity characteristics of the extracted gum, incorporated into printing paste at varying concentrations (1, 2, 3, and 4 g/ 50 ml of water), are presented in (Table 2; Fig. 3). The findings demonstrate that: (i) as gum concentration increases the apparent viscosity increases, (ii) an increase to 4 g of gum concentration yields a reduction in apparent viscosity, which appears to stabilize at higher shear rates, (iii) when the gum concentration is increased to 4 g, the apparent viscosity decreases and seems to stabilize at greater shear rates. Interestingly, apparent viscosity decreases as shear speeds rise.

**Scheme 1:**
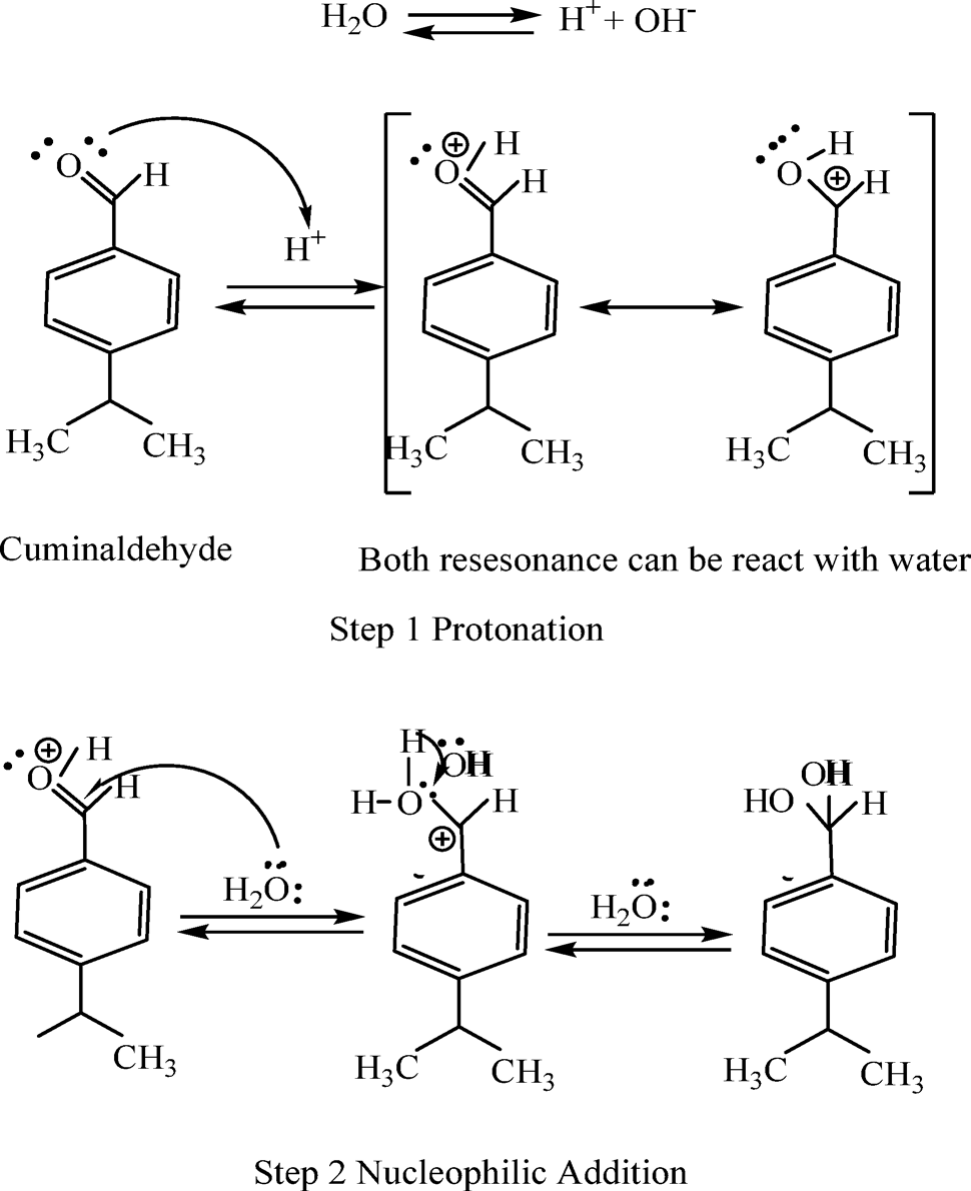
The interactions between the paste’s ingredients and the isolated natural gum.

As shown in Scheme 1, this phenomenon is probably due to the interactions between the paste’s ingredients and the natural gum that was removed. These interactions may help break down glycosidic bonds as the process goes on.

**Table 2:** How different amounts of natural gum affect the thickness of the printing paste.

Gum conc. %	1	2	3	4
RS cm^− 1^	Apparent Viscosity in centipoise			
3.4	4640	5464	840	590
6.8	3640	3980	770	590
10.2	2440	1816	582	370
13.6	1473	1440	420	328
17	1135	1484	290	290
20.4	1032	1400	269	260
23.8	771	1264	251	203
27.2	757	946	196	202
30.6	740	540	179	160
34	300	490	165	148

Figure 3 proves that the pastes we’re looking at have this non-Newtonian, pseudo-plastic ambiance to them. There’s a not-so-straightforward connection between shear stress and shear rate, which you can see in the overlapping flow curves when you go up and down. This means that when we check out the viscosity—basically how thick or thin the extracted gum behaves under heavy stress—it’s lower when we’re dealing with high shear rates compared to when we’re poking at the same paste with lower stress and slower rates. Plus, it’s note that these pseudo-plastic solutions don’t change over time^51,52^.

Table 3 shows how the extracted gum behaves under different pH levels—3, 7, and 9. The data indicate a clear pattern: as the shear rate and pH increase, the viscosity of the printing paste with the natural gum decreases. This is true, The viscosity of a printing paste formulated with natural gum diminishes as the shear rate and pH rise. This phenomenon is typical of a non-Newtonian, pseudoplastic fluid, in which viscosity declines with increased shear application. The reduction in viscosity is frequently linked to the disruption or deterioration of the gum molecules due to shear stress and/or alterations in the gum’s structure that are dependent on pH levels.

**Table 3:** Effects of pH levels on the apparent thickness of the printing paste made with extracted gum.

Printing paste pH	3	7	9
RS cm-1	Apparent Viscosity in centipoise		
3.4	1544	800	770
6.8	1530	730	728
10.2	1504	542	533
13.6	1482	380	332
17	1475	250	227
20.4	1428	229	225
23.8	1387	211	208
27.2	1381	156	146
30.6	1345	139	131
34	1225	125	111

**Fig. 4:**
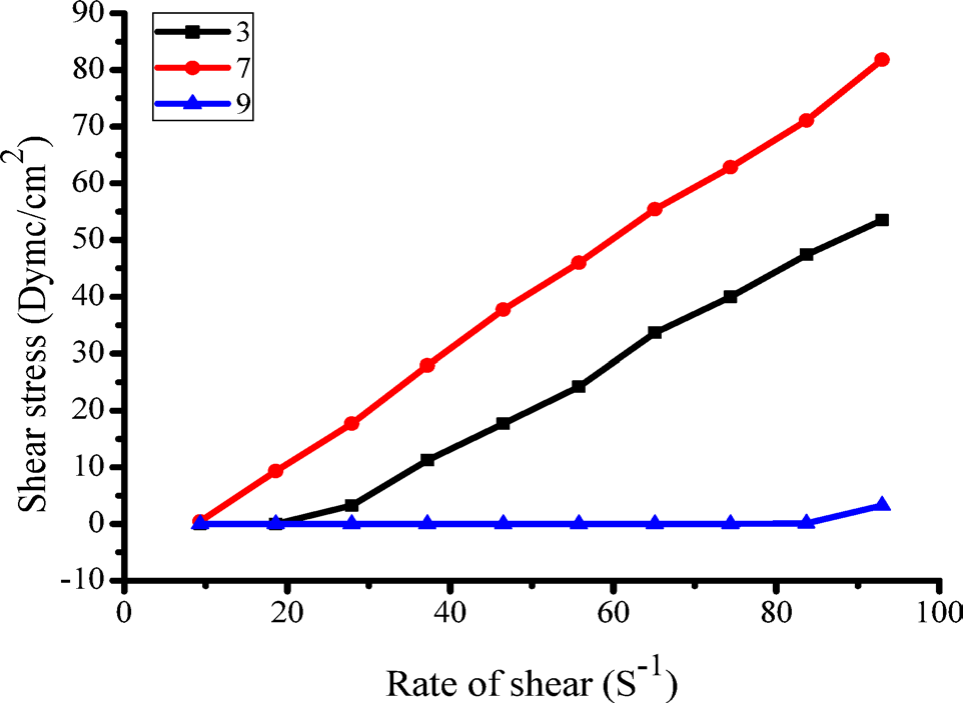
Shows how pH affects the flow characteristics of printing paste prepared from isolated natural gum.

In Fig. 4, the interaction between shear rate and shear stress is illustrated, providing insights into the behavior of the natural gum we have extracted. The graph features a notable hysteresis loop that originates at a shear rate of zero, ascends to a peak of 40, and subsequently descends back to zero. This observation supports the characterization of the gum as exhibiting pseudo-plastic, non-Newtonian properties, indicating that it becomes less viscous when stirred. The behavior is consistent across various pH levels, revealing distinct thixotropic characteristics. Furthermore, the rapid recovery of the gum’s internal structure after being deformed is closely associated with its thixotropic behavior, which we can quantify by analyzing the area between the ascending and descending curves on the graph .

### ANOVA test

The findings presented, including the correlation coefficient, variance analysis, and results from the ANOVA test, indicate that the conditions play a crucial role in the isolation processes. Additionally, the method of heating has a substantial impact on the rheological properties and viscosity of the printing paste. Furthermore, the data reflect that the isolation conditions considerably influence the characteristics of the gum derived from Aegle marmelous gum, as detailed in Table 4.

**Table 4:** The results of ANOVA test when applying isolated gum.

Source of variation	SS	df	MS	F	*p*-value	F crit
Rate of shear	3,234,346.423	15	187,872.4	10.2087327	5.42E -08	2.014804
Different amounts of isolated gum	1,668,223.553	2	854,167.4	35.02373617	1.42471E- 08	3.31583
Rate of shear	12,527,378	15	783,526.8	88.15843198	1.32E -20	2.014804
Different pH level	1,394,942	2	1,256,524	112.898042	1.09E- 14	3.31583

### Water absorbency and weight loss (Water Solubility)

**Fig. 5:**
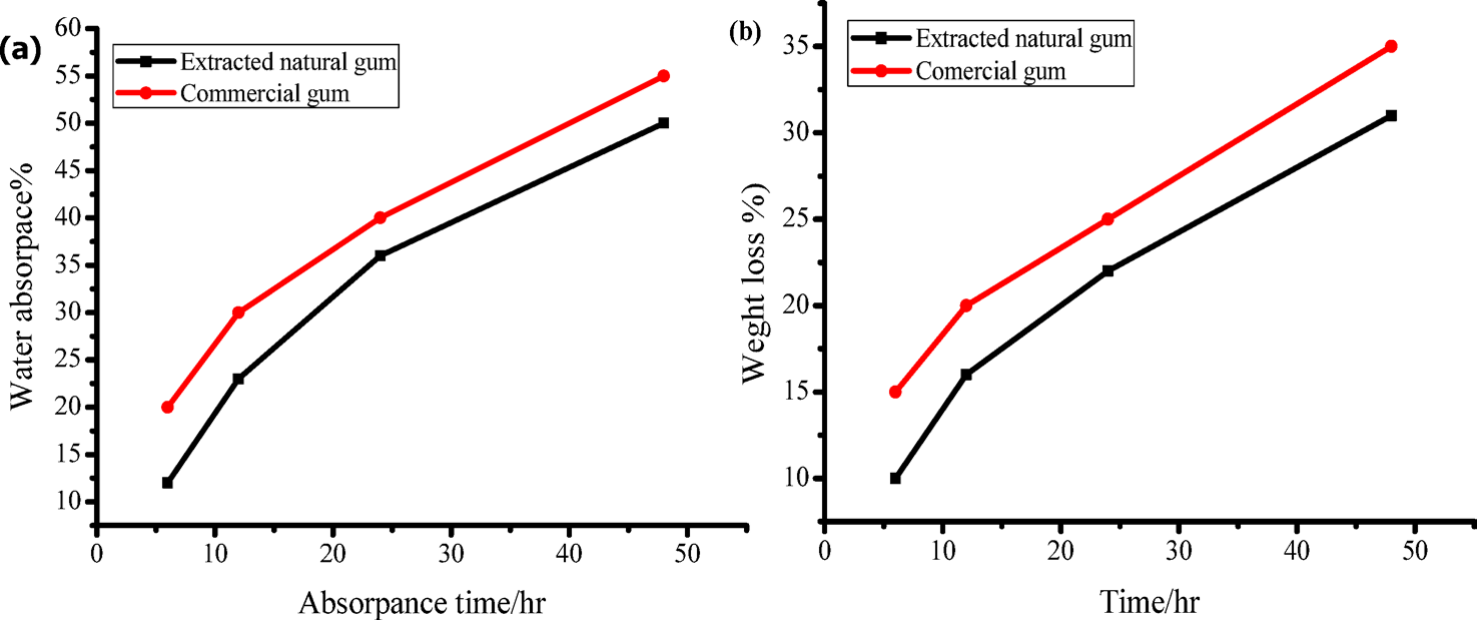
A comparison between the water absorbency (**a**) and weight loss (**b**) of the extracted gum and the commercial gum.

The weight loss and water absorption metrics for both commercial and extracted natural gums are shown in Figs. 5 (a) and (b). An examination of the data indicates that weight loss and water absorption increase proportionately with time. Interestingly, the extracted gum exhibits minimal weight loss and water absorption, in stark contrast to the commercial gum, which displays noticeably higher levels for both metrics. This may be because the extracted natural gum contains C = O groups, which help it effectively retain water molecules^56,57^.

### SEM (Scanning electron microscope)

**Fig. 6:**
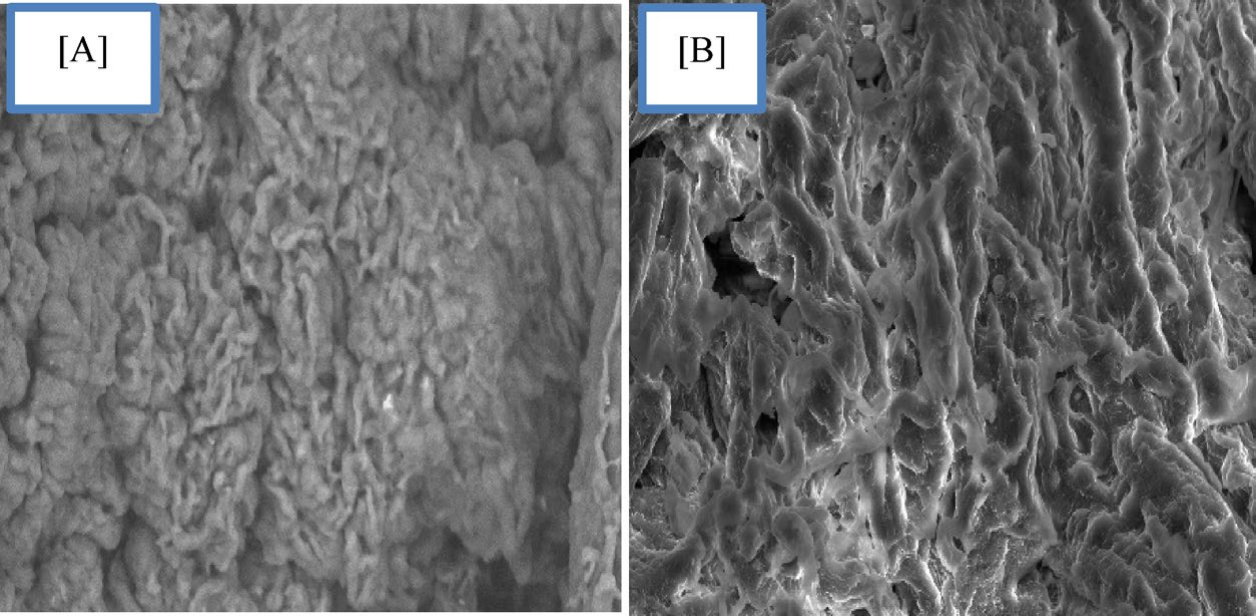
SEM (**A**) extracted natural gum from Aegle marmelous fruits assisted with MW irradiation, and (**B**) Commercial gum.

Characterization using SEM was done to determine any variations between the extracted and commercial gum to comprehend the morphology of the extracted natural gum (Fig. 6). Scanning electron microscopy (SEM) shows a slight difference between the two gums in terms of particle morphologies. While the commercial gum displayed more or less spherical aggregate particles, the extracted natural gum displayed elongation thread particles. This may be due to the influence of microwave radiation. The extraction procedures may be accelerated by the microwave heating approach, which would save time and energy. Elective molecular penetration by microwave radiation minimizes heat transfer problems and permits simultaneous heating. An electromagnetic field will rearrange the molecules of the gum that is created, causing ordered molecular movement. As a result, the molecular size and shape decrease, partitioning rises, and reaction velocity and mass transfer are improved.

### FTIR spectra of extracted natural gum and the commercial

**Fig. 7:**
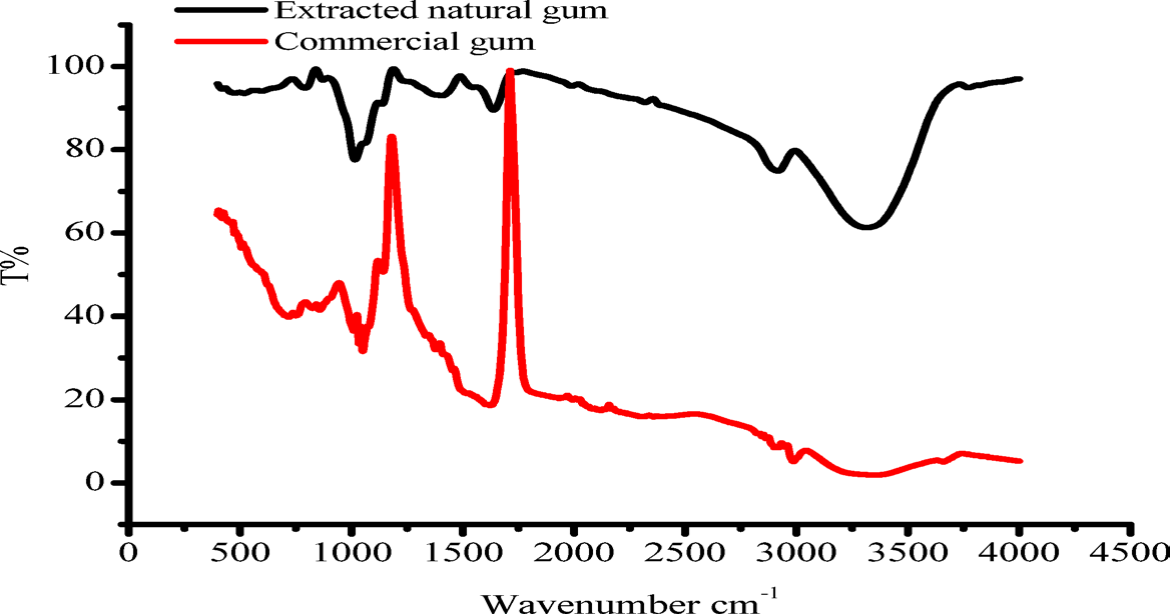
FTIR spectra of natural gum and commercial gum Features of printed textiles:

To compare the chemical structures of the extracted natural gum and the commercial gum, FTIR spectra were collected. Table 5 summarizes the distinctive IR wave number that was found. Figure 7 displays the FTIR spectra of the extracted natural gum and commercial gum. The O-H stretching modes are visible in the FTIR spectrum between 3341.07 cm^− 1^ and intensity 32.7239. The C-H stretching vibration of the commercial gum polymer and the water involved in hydrogen bonding caused the peak in the spectra at about 3000 cm^− 1^. The symmetrical CH_2_ group, primary C-OH alcoholic group, CH_2_OH, and CH_2_ twisting group were represented by the peaks in the spectra, which were 1612.2 cm^− 1^ with intensity 19.0558, 1137.8 cm^− 1^ with intensity 38.8073, 1053.91 cm^− 1^ with intensity 24.087, and 1024.02 cm^− 1^ with intensity 26.397, respectively. The band in the spectra close to 1650 cm^− 1^ was caused by associated water molecules. The –OH stretching group was represented by the sharpening of the absorption band around 3771.12 cm^− 1^ with an intensity of 94.967 in the extracted natural gum, the C-H group by the absorption band at 2917.77 cm^− 1^ with an intensity of 74.8812, and the C = O by the absorption band at 1991.14 cm^− 1^ with an intensity of 95.301. C-O, C-C, and secondary OH groups were represented by the absorption bands at 1547.59 cm^− 1^ with intensity 95.0061, 1407.78 cm^− 1^ with intensity 93.0202, and 1061.3 cm^− 1^ with intensity 77.5668, respectively. The range of 700–500 cm^− 1^ is known as polymer crystallinity^24^. The IR wave numbers of commercial gum and extracted gum are shown in Table 4; Fig. 7.

**Table 5:** Characteristic IR wave numbers of commercial gum and extracted natural gum from Aegle marmelous fruits.

Characteristic group	Wavenumber (cm^− 1^ )	Intensity	Characteristic group	Wavenumber (cm^− 1^ )	Intensity
	Commercial gum			Extracted natural gum	
O-H stretching vibration	3341.07	32.7239	OH- stretching	3771.12	94.967
C-H stretching of the CH_2_ group	2986.23	14.4815	C-H stretching	2917.77	74.8812
Symmetrical CH_2_ group	1612.2	19.0558	C = O	1991.14	95.301
C-OH primary alcoholic	1137.8	38.8073	C-O	1547.59	95.0061
CH_2_OH stretching mode	1053.91	24.087	C-C deformation	1407.78	93.0202
-CH_2_ twisting vibration	1024.02	26.397	2nd OH	1016.3	77.5668

### Characterization of printed fabrics

#### Fastness and color strength (K/S) characteristics

This study aimed to methodically assess and identify the most suitable printing material for creating prints that exhibit greater depth and remarkable fastness qualities. The investigation concentrated on evaluating color strength (K/S) alongside various fastness characteristics, which encompassed washing, perspiration, rubbing, and light fastness. These properties were examined across an array of printed textiles, notably cotton, wool, and polyester/cotton blends, using isolated gum as both a thickener and binder. This mixture was then heat-fixed at a temperature of 120 °C for 6 min. The findings from this investigation are compiled in Table 6. Table 6 offers a comparative analysis of color strength (K/S) and fastness properties across the various printed fabrics employing the extracted gum in its roles as thickener, binder, or in a combined capacity.

**Table 6:** Presents the properties related to (K/S) and fastness of various printed fabrics that have employed extracted gum in distinct roles, either functioning as a thickener, serving as a binder, or being utilized in a combined capacity (both functions are performed simultaneously).

Fabric	Gum used	K/S	Fastness								
			Washing		Perspiration				Rubbing		Light
					Acidic		Alkaline				
			Alt	St	Alt	St	Alt	St	Dry	Wet	
Cotton	Thickener	9.92	3–4	3–4	3–4	3–4	4–5	4–5	3–4	3–4	6–7
	Binder	7.73	3	3	3–4	3	3–4	4	3–4	3–4	6
	Both	1.21	2	3	2–3	2	2	2–3	2	2	3–4
Wool	Thickener	13.39	3–4	3–4	4	4	4–5	4–5	4	4	6–7
	Binder	10.76	3	3	3–4	3–4	3–4	4	3–4	3–4	6
	Both	3.03	2	3	2–3	2	2	2–3	2	2	4
Polyester/Cotton	Thickener	12.51	4	4	4	4	4	4	4	4	6–7
	Binder	8.91	3–4	3–4	4	4	4	4–5	3–4	3–4	6
	Both	1.93	2	3	2–3	2	2	2–3	2	2	3–4

The results shown in Table 6 provide several important insights: (a) all printed textiles have good wash fastness, ranging from 4 to 5; (b) the analysis of color difference between acidic and alkaline perspiration shows that the fastness characteristics are rated as good (3–4) to excellent (4–5); (c) an investigation into colorfastness to rubbing, using both wet and dry methods, shows that the results for these two approaches are consistent; (d) the use of extracted natural gum as a thickening agent in printed textiles produces exceptional light-fastness; (e) a comparative evaluation shows that printed wool and blend fabrics perform better in terms of color strength and fastness properties; (f) additionally, the use of separated gum as a thickener yields superior color strength and fastness characteristics compared to its use it as a binder.

Additionally, when compared to printed textiles that utilize only extracted natural gum (without either a binder or thickening agent), those printed fabrics incorporating extracted natural gum as both a binder and a thickening agent demonstrate weak color and fastness attributes.

A thickening agent serves to enhance the viscosity and plasticity of the printing paste, thereby inhibiting the migration of the dye beyond the designated design area. Conversely, a binder functions by creating a protective film encasing the pigment or dye, which secures its adherence to the fabric and guarantees its durability, ensuring resistance to factors such as fading and washing.So when we use the isolated gum as a thickener, it is necessary to add a binder, and vice versa, i.e., the isolated gum cannot be used as a binder or a thickener at the same time.

#### Impact of fixation type

**Fig. 8:**
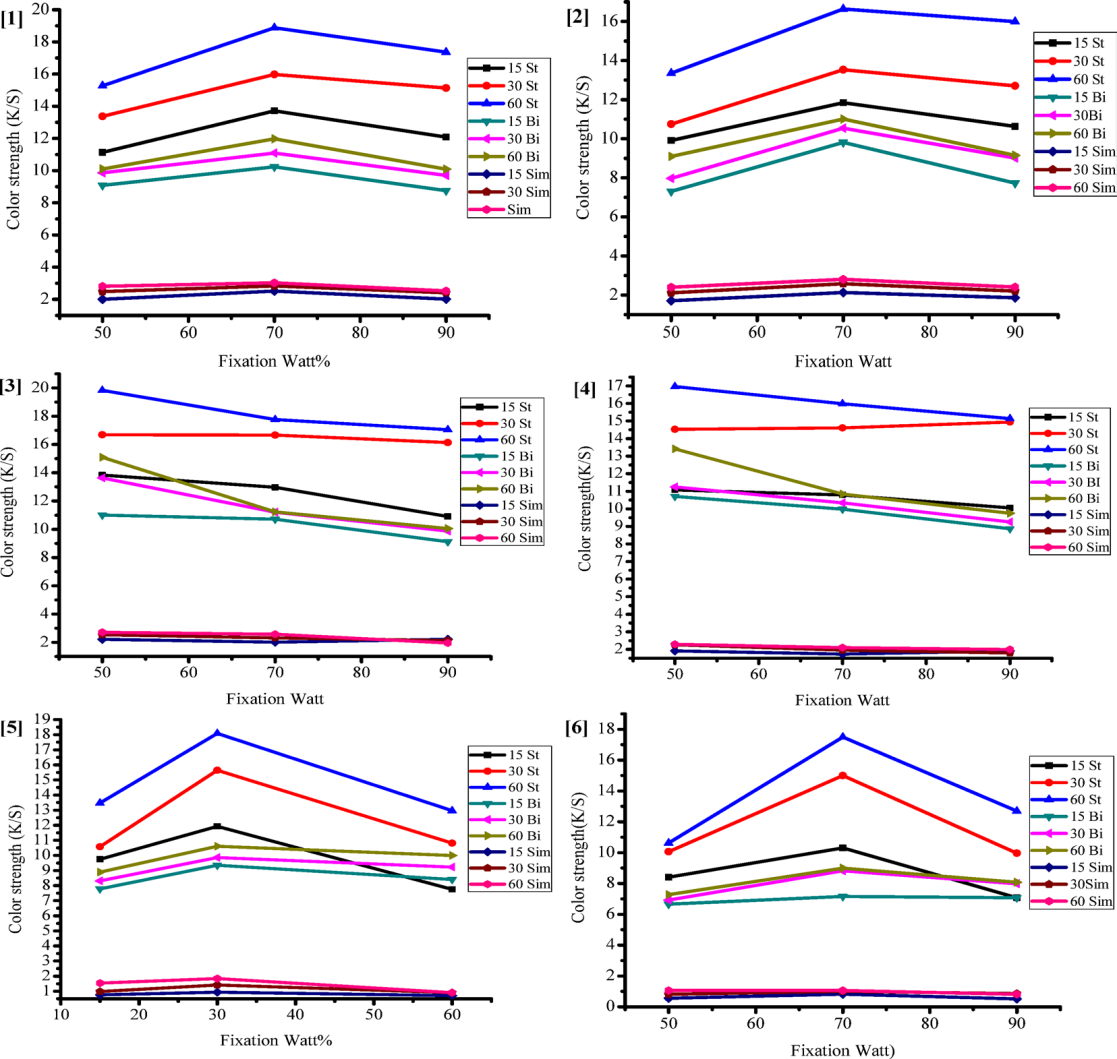
Impact of microwave fixing on printing of (1, 2) cotton, (3, 4) wool, and (5, 6) cotton/polyester blend fabrics (enclosed and closed), respectively at varying periods and power levels.

**Fig. 9:**
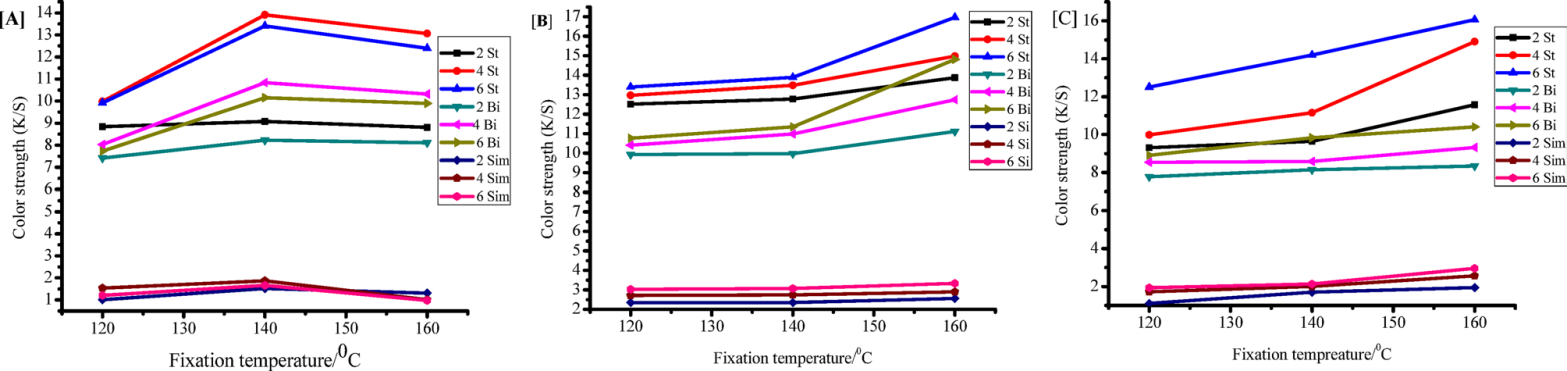
Impact of thermo fixation on printing of (**A**) Cotton, (**B**) Wool, and (**C**) polyester/Cotton blend fabrics at varying periods and temperatures.

In the present study, we used novel techniques that included microwave irradiation for 15–30, and − 60 s at power levels of 50–70, and − 90 watts using both closed and enclosed systems (Fig. 8). For comparison we subjected the printed samples to standard thermo fixation methods for 2–4, and − 6 min at 120, 140, and − 160 °C (Fig. 9). After the fixation process, we let the samples dry at room temperature, cleaned them using the methods described in the experimental section, and then assessed their color yield (K/S) and other fastness characteristics, such as light fastness, washing, rubbing (dry and wet), and perspiration (in both acidic and alkaline conditions).

Figure 8 shows a clear link between the K/S values of all the printed fabrics and how long they’re exposure to microwave irradiation. The longer the microwave time, the stronger the color gets. You see a similar trend with microwave power, but it depends on the fabric structure and the type of gum used. The highest K/S value happened at 70 watts, while it dropped to a low at 90 watts for printed cotton and cotton/polyester blends. Interestingly, printed wool fabric hit its sweet spot at 50 watts for 60 s. The results also reveal that closed samples show a notable jump in K/S values, no matter how long they’re exposed to the microwave or the intensity. This is mainly because microwaves heat things by using water (a polar solvent), which absorbs energy and gets hot. When you seal those printed samples, less water evaporates compared to the open ones, meaning more heat builds up on the fabric. This thermodynamic effect strengthens the connection between water molecules on the printed surface and the K/S values.

On the flip side, the info in Fig. 8 shows that when you crank up the fixation temps and times (thermo fixation), the K/S values usually go up, but not when it comes to printed cotton. The peak K/S values popped up at 160 °C for 6 min for both wool and polyester/cotton blends, while cotton hit its sweet spot at 140 °C for the same time (Fig. 9).

Looks like using microwave irradiation is a solid way to set pigment prints on different types of fabrics. The K/S value, which basically measures how vibrant the color is, really depends on how powerful the microwave is and how long you expose the fabric. Interestingly, when you compare the K/S values, the fabrics that got fixed with microwave treatment generally show better results in closed samples than in enclosed ones. Plus, if you use natural gum as a binder, it gives you a better K/S value than if it’s just acting as a thikening agent. On the flip side, if you use the natural gum without any extra stiffening or thickening stuff, it ends up giving the lowest K/S value.

### Performance of printing fabric

Table 7 delineates the performance metrics associated with the printed fabric, which encompass add-on, color strength (K/S), color uniformity, dye penetration, and fixation percentage. During this stage, the natural gum extracted serves dual purposes as both a binder and thickening agent, as outlined in the experimental procedures. After this application, the fixation occurs via microwave exposure at 70 watts for cotton and polyester/cotton blend fabrics, while a lower power setting of 50 watts is employed for wool. The duration allocated for the fixing process across all printed fabric types is standardized at 60 s.

The differences in color yield, penetration percentage, and fixation percentage across the fabrics studied can be attributed to variations in their chemical compositions and fabric characteristics, as well as the influence of microwave irradiation. The data presented in Table 7 indicates that the use of extracted natural gum as a thickening agent results in superior performance in these metrics compared to its application as a thickening agent. Furthermore, the implementation of pulsed microwave irradiation serves to regulate temperature and alleviate exothermic reactions by promoting rapid interactions between the fabric and pigment. This facilitates a stable temperature environment throughout the fixation phase, enhancing the overall effectiveness of the dyeing process.

**Table 7:** The printing performance of the microwave-irradiated printed fabrics (enclosed samples) was compared with commercial alternatives.

Used gum	K/S	Penetration%	Unevenness	Stiffness	F%
Cotton					
Thickener	18.88	73	11.7181	1731.6	83.21
Com thickener	18.09	71	11.0242	1710.9	83.00
Binder	11.98	60	9.35	1228.9	78.83
Com. binder	10.93	61	9.72	1204.3	79.1
Wool					
Thickener	19.83	95	15.8326	1667.4	95.89
Com thickener	18.79	95	14.9549	1633.9	96.91
Binder	15.09	83	11.0437	1351.7	91.99
Com. binder	15.13	82	11.0001	1307.6	90.72
Polyester/cotton					
Thickener	18.09	72	12.4017	1828.1	87.3
Com thickener	17.99	72	12.9009	1747.9	86.93
Binder	10.61	65	9.90	1447.7	82.8
Com. binder	10.11	63	8.8087	1374.9	80.00

### Fastness properties

Table 8 shows the color fastness results for printed fabrics that went through either microwave treatment or thermal fixing. The findings reveal that the samples fixed with thermal methods had better fastness compared to those treated with microwaves. This makes sense since using microwaves is usually linked to better color durability. Also, it’s worth noting that using separated gum as a binder led to the best outcomes for color fastness.

**Table 8:** Properties of color fastness for the printed samples treated with microwave irradiation and thermal fixation methods.

Gum used	Fastness properties								
	Washing		Rubbing		Perspiration				Light
					Acidic		Alkaline		
	Alt	St	Dry	Wet	Alt	St	Alt	St	
Microwave fixation									
Cotton									
Thickener	3–4	3–4	4	3–4	3–4	3–4	4	4	6
Binder	3	2–3	2–3	3	3	3	3	3	4–5
Wool									
Thickener	4–5	4–5	4	4	4	4	4	4	6–7
Binder	3–4	3–4	3–4	3–4	3–4	3–4	3–4	3	5–6
Polyester/cotton									
Thickener	3–4	3–4	3–4	4	4	4	3–4	3–4	5–6
Binder	3	3	3	3	3–4	4	4	3	5
Thermo fixation									
Cotton									
Thickener	3	3	3	3	3	3	3	3	6
Binder	2–3	2–3	3	2–3	2–3	2–3	2–3	2–3	5–6
Wool									
Thickener	3	3	3–4	3–4	3–4	3–4	4	4	5–6
Binder	2–3	2–3	3	3	3	3	2–3	2–3	6
Polyester/Cotton									
Thickener	3–4	3–4	4	4	4	4	4–5	3–4	6
Binder	3	3	3	2–3	3	3	3	3	5

Table 9 illustrated a comparison between isolated and the commercial gum.

**Table 9:** A comparison between isolated and commercial gum.

Comparison	Commercial	Isolated
Weight loss	High	Low
H2O absorbance	High	Low
Particle morphologies	Spherical aggregate	Elongation thread particles
Rheological	non-Newtonian, pseudoplastic fluid	non-Newtonian, pseudoplastic fluid
Fastness	Very good	Very good-excellent
K/S	Low	High
Penetration%	Low	High
Unevenness	Low	High
Stiffness	Low	High
F%	Low	High

### Analysis of biodegradability processes

**Fig. 10:**
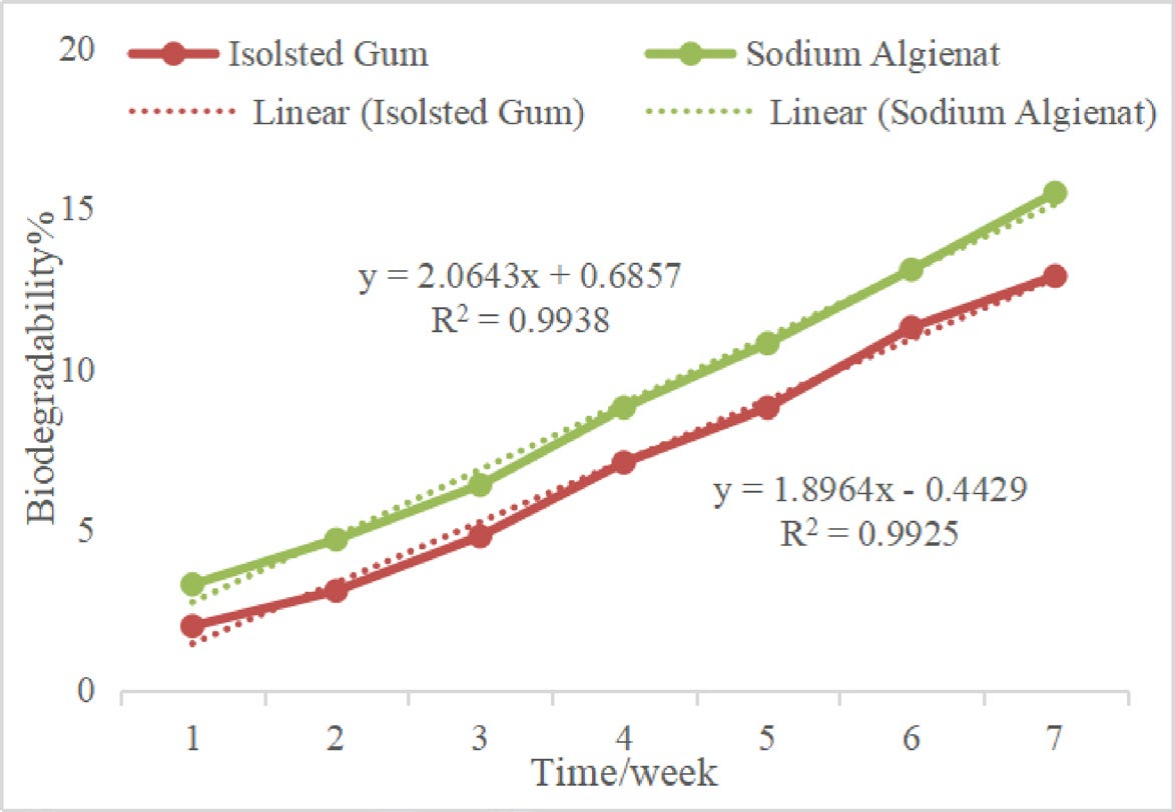
The percentage of biodegradability observed for both isolated and commercial gums was assessed over three weeks in conditions simulating burial within soil or compost.

Figure 10 illustrates the experimental findings regarding the biodegradability of various gum samples over three weeks, utilizing the soil/compost burial method. The data indicate that, after a 3-week duration, the biodegradability rates for isolated and commercial gum were 12.91% and 15.53%, respectively. To ensure accurate predictions of biodegradability over time, linear regression models were employed. The analysis revealed a strong linear correlation (R² > 0.9896 for isolated gum and R² > 0.9909 for the commercial gum) between biodegradability percentage and time, as depicted in Fig. 10. It is worthy said that The corresponding linear equations suggest that commercial gum is expected to fully biodegrade within approximately 2.23 years (or 24.3 months), which is significantly shorter than the 2.00 years (or 24 months) estimated for the isolated gum.

## Conclusion

This study focused on whether the natural gum isolated from the Aegle marmelous fruit can be used in textile printing paste for stuff like thickening agents and binders. The result indicated that the rheological properties of the paste containing the isolated natural gum were unique. The printing paste showed a noticeable boost in apparent viscosity, and this effect didn’t seem to depend on how much gum they used or the pH levels. Plus, the pastes showed some non-Newtonian pseudo-plastic behavior, meaning the relationship between shear stress and shear rate wasn’t straightforward, and the flow curves sort of lined up nicely when going up and down. When they compared it to commercial thickening agents, the natural gum from Aegle marmelous had way less weight loss and water absorption. And get this—fabrics printed with this gum, used as both a thickening agent and binder, looked way better in terms of color brightness and lasted longer than those printed with other natural gums. Before comparing it to samples done with standard methods, the printed fabrics got a fix with microwave irradiation for different times and power levels. They discovered that using microwave radiation was super effective for reviving different printed fabric surfaces. Before the comparative analysis with the attached samples utilizing the established protocol, the printed samples underwent a fixing process through microwave irradiation, with variations in both duration and wattage. The findings indicate that different printed fabric surfaces can be successfully fixed through the application of microwave radiation.

## Data Availability

Data is provided within the manuscript.

## References

[1] El-Rahman, A. et al. Advancements in thickening agents used in textile printing. *Egypt. J. Chem.* **65** (1), 565–579. 10.21608/ejchem.2021.83272.4088 (2022).

[2] Bajaj, P. et al. Synthetic thickeners in textile printing: a critique. *J. Macromolecular Sci. Part. C: Polym. Reviews*. **33** (3), 321–348. 10.1080/15321799308021439 (1993).

[3] Jassal, M. et al. Acrylic-based thickeners for pigment printing—a review. *J. Macromolecular Sci. Part. C: Polym. Reviews*. **42** (1), 1–34. 10.1081/MC-120003093 (2002).

[4] Abdelrahman, M. et al. Review in textile printing technology. *Egypt. J. Chem.* **63** (9), 3465–3479. 10.21608/ejchem.2020.23726.2418 (2020).

[5] Saad, F. et al. A valuable observation on thickeners for valuable utilisation in the printing of different textile fabrics. *Egypt. J. Chem.* **65** (4), 431–448. 10.21608/ejchem.2021.96612.4521 (2022).

[6] Hossain, M. M. et al. Advancements of eco-friendly natural antimicrobial agents and their transformative role in sustainable textiles. *SPE Polym.*10.1002/pls2.10135 (2024).

[7] Veena, H., Shanmugam, K. A. & Review of Textile Fabrication Using Bioactive Compounds Derived from Natural Products. Proceedings of Anticancer Research. 7(6):7–12 (2023). 10.26689/par.v7i6.5162

[8] Morais, D. S., Guedes, R. M. & Lopes, M. A. Antimicrobial approaches for textiles: from research to market. *Materials* **9** (6), 498. 10.3390/ma9060498 (2016). 28773619 10.3390/ma9060498 PMC5456784

[9] Bibi, A. et al. Synthetic vs. natural antimicrobial agents for safer textiles: a comparative review. *RSC Adv.* **14** (42), 30688–30706. 10.1039/D4RA04519J (2024). 39328870 10.1039/d4ra04519j PMC11425080

[10] Marathe, S. J. et al. Improvements in the extraction of bioactive compounds by enzymes. *Curr. Opin. Food Sci.* **25**, 62–72. 10.1016/j.cofs.2019.02.009 (2019).

[11] Manasa, D. et al. Enzyme-assisted extraction of bioactive compounds from ginger (Zingiber officinale Roscoe). *Food Chem.* **139** (1–4), 509–514. 10.1016/j.foodchem.2013.01.099 (2013). 23561138 10.1016/j.foodchem.2013.01.099

[12] Sanjeewa, K. A. et al. Enzyme-assisted extraction of bioactive compounds from seaweeds and microalgae. *TRAC Trends Anal. Chem.* **1**, 117266. 10.1016/j.trac.2023.117266 (2023).

[13] Boulila, A. et al. Enzyme-assisted extraction of bioactive compounds from Bay leaves (Laurus nobilis L). *Ind. Crops Prod.* **74**, 485–493. 10.1016/j.indcrop.2015.05.050 (2015).

[14] Łubek-Nguyen, A. et al. Application of enzyme-assisted extraction for the recovery of natural bioactive compounds for nutraceutical and pharmaceutical applications. *Appl. Sci.* **12** (7), 3232. 10.3390/app12073232 (2022).

[15] Rahaman, M. T., Pranta, A. D., Repon, M. R., Ahmed, M. S. & Islam, T. Green production and consumption of textiles and apparel: importance, fabrication, challenges and future prospects. *J. Open. Innovation: Technol. Market Complex.* 100280. 10.1016/j.joitmc.2024.100280 (2024).

[16] Pranta, A. D., Rahaman, M. T., Repon, M. R. & Shikder, A. A. R. Environmentally sustainable apparel merchandising of recycled cotton-polyester blended garments: analysis of consumer preferences and purchasing behaviors. *J. Open. Innovation: Technol. Market Complex.* **10** (3), 100357. 10.1016/j.joitmc.2024.100357 (2024).

[17] Rahaman, T. & Khan, S. H. Green merchandising of textiles and apparel in a circular economy: recent trends, framework, challenges and future prospects towards sustainability. *J. Open. Innovation: Technol. Market Complex.* 100457. 10.1016/j.joitmc.2024.100457 (2024).

[18] Rahaman, M. T. & Hossain Khan, M. S. Biomaterials for manufacturing environmentally sustainable textiles and apparel: sources, applications, challenges, enablers and future directions. *Int. J. Environ. Sci. Technol.* 1–56. 10.1007/s13762-025-06481-7 (2025).

[19] Rahaman, M. T., Pranta, A. D. & Ahmed, S. Transitioning from industry 4.0 to industry 5.0 for sustainable and additive manufacturing of clothing: framework, case studies, recent advances, and future prospects. *Mater. Circular Econ.* **7** (1), 1–37. 10.1007/s42824-025-00176-7 (2025).

[20] Khan, M. S. H., Rahaman, M. T., Pranta, A. D. & Hasan, M. K. Eco-friendly organic nanomaterials for multifunctional textiles: sources, applications, recent advances and future prospects towards sustainability. *Int. J. Environ. Sci. Technol.* 1–58. 10.1007/s13762-024-06299-9 (2025).

[21] Pranta, A. D., Rahaman, M. T., Ahmed, M. S. & Arefin Rafi, M. S. Navigating eutrophication in aquatic environments: Understanding impacts and unveiling solutions for effective wastewater management. *Res. Ecol.* **5** (3), 11–18. 10.30564/re.v5i3.5908 (2023).

[22] Marathe, S. J. et al. Improvements in the extraction of bioactive compounds by enzymes. *Curr. Opin. Food Sci.* **25**, 62–72. 10.1016/j.cofs.2019.02.009 (2019).

[23] Sobh, N. et al. New insights into the role of color extraction from (Aegle Marmelos leaf) using a non-traditional heating source. *Pigm. Resin Technol.* **54** (1), 53–64. 10.1108/PRT-05-2023-0041 (2024).

[24] Bisma, A. S. et al. Green extraction of colorants from achiote seeds for dyeing mordanted nylon fabric. *Surf. Innovations*. **25**, 1–0. 10.1680/jsuin.24.00073 (2024).

[25] Buyukakinci, B. Y. & Karadag, R. Optimization of the natural dyes extraction (Madder and Wallonia oak) and cotton dyeing using microwave irradiation. *Text. Leather Rev.* **5**, 451–462. 10.31881/TLR.2022.42 (2022).

[26] Ben Ticha, M. et al. Bark residues as a natural colorant based on response surface methodology: A challenging approach to a sustainable dyeing process for acrylic fabrics. *Sustainability* **14** (7), 4134. 10.3390/su14074134 (2022).

[27] Kumar, R. et al. Process optimization for extraction of natural dye from M. Philippinensis fruits and its application on different fabrics. *World J. Pharm. Res.* **5** (4), 927–945. 10.20959/wjpr20164-5876 (2016).

[28] Naqvi, S. A. et al. Modern ecofriendly approach for extraction of Luteolin natural dye from weld for silk fabric and wool yarn dyeing. *Sustainable Chem. Pharm.* **39**, 101554. 10.1016/j.scp.2024.101554 (2024).

[29] Manicketh, T. J. & Francis, M. S. Extraction of natural colorants from Araucaria columnaris, Macaranga peltata and Averrhoa bilimbi for textile coloration. *Int. J. Cloth. Sci. Technol.* **32** (6), 789–801 (2020). https://www.emerald.com/insight/0955-6222.htm

[30] Khanal, A. et al. Bael (Aegle marmelos), an underutilized fruit with enormous potential to be developed as a functional food product: a review. *J. Food Process. Preserv.* **2023** (1), 8863630. 10.1155/2023/8863630 (2023).

[31] Sharma, N. et al. Aegle marmelos (L.) correa: an underutilized fruit with high nutraceutical values: a review. International Journal of Molecular Sciences. Sep 17;23(18):10889 (2022). 10.3390/ijms231810889 10.3390/ijms231810889 PMC9504793 36142805

[32] Baliga, M. S. et al. Phytochemistry and medicinal uses of the Bael fruit (Aegle Marmelos Correa): A concise review. *Food Res. Int.* **44** (7), 1768–1775. 10.1016/j.foodres.2011.02.008 (2011).

[33] Charoensiddhi, S. & Anprung, P. Bioactive compounds and volatile compounds of Thai Bael fruit (Aegle Marmelos (L.) Correa) as a valuable source for functional food ingredients. *Int. Food Res. J.* **15** (3), 287–295 (2008).

[34] Sarkar, A. et al. Phytochemicals and nutritional constituent evaluation of Bael (Aegle marmelos) fruit pulp at different development stage. *Asian Food Sci. J.* **20** (1), 78–86. 10.9734/afsj/2021/v20i130257 (2021).

[35] Krushna, G. S. S. et al. Aegle marmelos fruit extract attenuates isoproterenol-induced oxidative stress in rats. *J. Clin. Biochem. Nutr.* **50** (3), 199–204. 10.3164/jcbn.11-69 (2012). 22573921 10.3164/jcbn.11-69 PMC3334372

[36] Wakade, A. S. et al. Protective effect of Piper longum fruits against experimental myocardial oxidative stress induced injury in rats. *J. Nat. Remedies Jan*. **1**, 43–50 (2009). http://www.jnronline.com

[37] Baliga, M. S., Thilakchand, K. R., Rai, M. P., Rao, S. & Venkatesh, P. Aegle marmelos (L.) Correa (Bael) and its phytochemicals in the treatment and prevention of cancer. *Integr. cancer Ther.* **12** (3), 187–196. 10.1177/1534735412451320 (2013). http://ict.sagepub.com 23089553 10.1177/1534735412451320

[38] Devi Sampath, P. & Vijayaraghavan, K. Cardioprotective effect of α-mangostin, a Xanthone derivative from mangosteen on tissue defense system against isoproterenol‐induced myocardial infarction in rats. *J. Biochem. Mol. Toxicol.* **21** (6), 336–339. 10.1002/jbt.20199 (2007). 17994576 10.1002/jbt.20199

[39] Sampath, P. D. & Vijayaragavan, K. Ameliorative prospective of alpha-mangostin, a Xanthone derivative from Garcinia mangostana against β-adrenergic cathecolamine-induced myocardial toxicity and anomalous cardiac TNF-α and COX-2 expressions in rats. *Exp. Toxicol. Pathol.* **60** (4–5), 357–364. 10.1016/j.etp.2008.02.006 (2008). 18424012 10.1016/j.etp.2008.02.006

[40] Kamalakkannan, N. & Stanely, M. P. Effect of Aegle marmelos Correa.(Bael) fruit extract on tissue antioxidants in streptozotocin diabetic rats. 15332498

[41] Jindal, M. et al. Exploring potential new gum source Aegle marmelos for food and pharmaceuticals: physical, chemical and functional performance. *Ind. Crops Prod.* **45**, 312–318. 10.1016/j.indcrop.2012.12.037 (2013).

[42] Roiy, A. et al. The structure of Bael (Aegle marmelos) gum. *Carbohydr. Res.* **54** (1), 115–124. 10.1016/S0008-6215(00)80560-1 (1977).

[43] Jindal, M. et al. Aegle marmelos fruit pectin for food and pharmaceuticals: Physico-chemical, rheological and functional performance. *Carbohydr. Polym.* **93** (2), 386–394. 10.1016/j.carbpol.2012.12.012 (2013). 23499073 10.1016/j.carbpol.2012.12.012

[44] Pranta, A. D. & Rahaman, M. T. Extraction of eco-friendly natural dyes and biomordants for textile coloration: a critical review. *Nano-Structures Nano-Objects*. **39**, 101243. 10.1016/j.nanoso.2024.101243 (2024).

[45] Allam, O. G. et al. Synergistic effect of alkali treatment and microwave irradiation on the dyeing properties of polyester–wool blend fabrics. *Int. J. Sci. Res.* **4** (9), 1697–1705 (2015).

[46] Haggag, K. et al. Recycling of waste PET into useful alkyd resin synthesis by microwave irradiation and applied in textile printing. *Res. J. Text. Appar.* **18**, 1: 80–88. 10.1108/RJTA-18-01-2014-B010 (2014).

[47] Rafat, B. et al. Industrial scale production of resist/discharge printed cotton knitted garments using bio-technique. Journal of Applied Sciences Research, 9, No. 1, 163–169 ref. 20 (2013). http://www.aensiweb.com/jasr/jasr/2013/163-169.pdf

[48] Rahaman, M. T., Hasan, M. K. & Khan, M. S. H. Environmental impact measurement and chromatic performance evaluation of denim washing: a comparison to conventional and sustainable approaches for cleaner production. *Environ. Sci. Pollut. Res.* **32** (10), 6110–6129. 10.1007/s11356-025-36099-8 (2025). 10.1007/s11356-025-36099-8 39976792

[49] Rahaman, M. T., Shikder, A. A. R. & Al Mamun, M. A. Environmentally sustainable color fading approaches of denim fabric using alternative garments dry process: an insight into chromatic parameters and physical properties. *J. Open. Innovation: Technol. Market Complex.* **10** (4), 100435. 10.1016/j.joitmc.2024.100435 (2024).

[50] Elshemy, N. et al. Employing ultrasonic waves to extract flax seed for textile printing and applying creative fashion designs. *Res. J. Text. Appar.* **29** (2), 233–253. 10.1108/RJTA-12-2022-0148 (2025).

[51] Adeel, S. et al. Microwave-assisted sustainable dyeing of wool fabric using cochineal-based carminic acid as natural colorant. *J. Nat. Fibers*. **16** (7), 1026–1034. 10.1080/15440478.2018.1448317 (2018).

[52] Adeel, S. et al. Microwave-Supported green dyeing of mordanted wool fabric with Arjun bark extracts. *J. Nat. Fibers*. **18** (1), 136–150. 10.1080/15440478.2019.1612810 (2019).

[53] Rehan, M. et al. Concurrent dyeing and finishing of textile fabrics using chemically modified peanut red skin extract. *Fibers Polym.* **24**, 2357–2365. 10.1007/s12221-023-00195-8 (2023).

[54] Elshemy, N. S. et al. Natural colourants from Sargassum polycystum c.agardh (Phaeophyceae) for fabric dyeing: an eco-friendly method for textile colouring by response surface methodology. *Appl. Phycol.* **5** (1), 143–157. 10.1080/26388081.2024.2399179 (2024).

[55] El-Shemy, N. et al. Synthesis and applications of nano binder based on plant oils. Journal of natural fibers. Jan 2;14(1):10–25 (2017). 10.1080/15440478.2015.1133364

[56] Hebash, A., Abdelrahman, A., Nassar, S., Elsayad, H. & Elshemy, N. Microstructural features of Galactomannan Fenugreek gum newly oxidized by sodium perborate under microwave irradiation for reactive printing. Egyptian Journal of Chemistry, 62(11), pp.1971–1986(2019). 10.21608/ejchem.2019.5236.1466

[57] Hebeish, A., Abdelrahman, A., Nassar, S., Elsayad, H. & Elshemy, N. Utilization of the combined effect of ultrasound waves and sodium metaperiodate in preparation of oxidized galactomannan fenugreek gum for medical and other purposes. Egyptian Journal of Chemistry, 62(Special Issue (Part 2) Innovation in Chemistry), pp.627–644 (2019). 10.21608/ejchem.2019.17500.2075

